# Gender Inequities in the Multiple Sclerosis Community: A Call for Action

**DOI:** 10.1002/ana.25359

**Published:** 2018-11-30

**Authors:** International Women in MS

We thank Moneim and colleagues for calling attention to the issue of gender inequality in MS clinical trial steering committees and in authorship on publications emerging from phase 3 clinical trials.^1^ Gender inequality is a long‐standing problem in publication practices in general,^2^ as well as in neurology,^3^, ^4^ academic medicine,^5^ and science,^6^ so it is not surprising to see it reflected in the field of multiple sclerosis (MS). This occurs despite the availability of a substantial number of successful senior female academic neurologists and neuroscientists worldwide who have extensive experience in MS clinical and basic research methodology; some are cosigners of this letter. Are there additional reasons beyond those articulated by Moneim and colleagues to explain why senior female neurologists are not approached to play lead roles in MS clinical trial committees and more broadly, clinical MS neurologists and MS researchers?

Moneim and colleagues observed that women have been historically underrepresented as first and last authors on publications from phase 3 clinical trials in MS sponsored by pharmaceutical companies, which tend to repeatedly choose a select group of academicians to serve as authors.^7^ In addition, eminent lead investigators have to some extent, consciously or subconsciously, nurtured a closed group for clinical trial leadership in which women are not well represented. Publication practices may also be influenced by gender balance on journal editorial boards. In an informal examination of chief and section editors of 17 major journals that publish MS studies, we found that median representation by women was 16.6%. This is approximately half of the proportion of female faculty in the 29 top‐ranked US academic neurology programs (30.8%).^4^ Gender imbalance also occurs in scientific programs at professional meetings^8^ and in recognition awards,^9^ issues that have been addressed with limited success by the American Neurological Association, American Academy of Neurology, and the American and European Committees for Treatment and Research in MS.

Gender discrimination is costly, not only from a personal career perspective, but because it excludes or delays important contributions from skilled, talented individuals. This can affect progress toward a better understanding of the pathogenesis and treatment of neurological diseases, including MS.

Although it is disheartening to see continuing evidence of the gender discrimination that permeates not only academic medicine and science, but also society at large, we are encouraged that there is interest in shedding greater light on the issue. We believe that it is time for the MS community to work harder toward achieving equal participation by all women at all levels. To this end, we submit this letter as a call to action, and offer the following recommendations:

1. Develop structured opportunities to foster greater awareness of gender‐based imbalances and existing biases, including implicit or unconscious bias. This can occur on a committee level in professional organizations and should include representatives from pharmaceutical companies. The primary goal would be to establish task forces charged with gathering and analyzing data on gender concerning:
  a Clinical trial leadership, including clinical trial steering committees, data safety monitoring boards, authorship committees, and other active committees.
  b Participation by physicians and basic scientists on editorial boards and grant review panels and in scientific programs, professional organizations, and academic institutions, including neurology departments, as well as in other relevant entities.
2. Establish specific goals toward achieving equal representation by women at all levels in a timely fashion.
  a Develop mechanisms to track and regularly review progress toward these goals, with an ultimate goal of achieving and maintaining gender parity.
  b Create mechanisms to report inequalities and encourage continued efforts to raise awareness.
  c Develop mechanisms to recognize and celebrate contributions from women.
  d Promote efforts to support and mentor women (such as the International Women in MS, the Women Leading in Neurology leadership program of the American Academy of Neurology,^3^ and the Association for Women in Science), especially for junior investigators.

There are many models and transformative voices^3^, ^6^, ^10^, ^11^, ^12^ that can be tapped in efforts to swing the pendulum toward a truly equal opportunity environment in the MS community. It is clear that conscious, thoughtful, and persistent efforts by all stakeholders, both men and women, are needed to implement enduring change worthy of celebration. We look forward to participating in an ongoing and collaborative dialogue toward achieving this important goal.

This letter was authored by Emmanuelle Waubant, Lilyana Amezcua, Nancy Sicotte, Kerstin Hellwig, Lauren Krupp, Bianca Weinstock‐Guttman, Ann Yeh, Robyn M. Lucas, Erin E. Longbrake, Vijay Yadav, Mary Rensel, Soe Mar, Carrie Hersh, Valerie Block, Frauke Zipp, May H. Han, Rebecca Spain, Eve E. Kelland, Leigh Charvet, Dalia Dimitri, Caroline Papeix, Anne H. Cross, Matilde Inglese, Maria Pia Amato, Laura Airas, Emmanuelle Leray, Maria Pia Sormani, Anneke Van der Walt, Sandra Vukusic, Tamara Castillo‐Trivino, Silvia Tenembaum, Olga Ciccarelli, Giulia Bommarito, Maria Petracca, Elisabeth Gulowsen Celius, Monica J. Carson, Le H. Hua, Ingrid Van der Mei, Catherine Lubetzki, Vilija Jokubaitis, Maria Trojano, Rhonda Voskuhl, Mar Tintore, Hanne Harbo, Nasrin Asgari, Laura Piccio, Jodie M. Burton, Helen Tremlett, Myla D. Goldman, Laure Michel, Yunyan Zhang, Riley Bove, Jacqueline A. Quandt, Fiona Costello, Carolina Ionete, Christine Lebrun‐Frenay, Julia Pakpoor, Carolyn Bevan, Sarah A. Morrow, Amy T. Waldman, Jiwon Oh, Dina Jacobs, Jacqueline Palace, Ruth Ann Marrie, Seema K. Tiwari‐Woodruff, Luanne M. Metz, Rosa Cortese, Tanuja Chitnis, Leslie Benson, Etty (Tika) Benveniste, Jill Conway, Ilana Katz Sand, Jennifer Orthmann Murphy, Mariko Kita, Claire Riley, Joan M. Goverman, Annette M. Langer‐Gould, Christina J. Azevedo, Idanis Berrios Morales, Lisa F. Barcellos, Elizabeth Crabtree, Prue Plummer, Afsaneh Shirani, Katherine Whartenby, Fabienne Brilot‐Turville, Elaine Kingwell, Patricia Coyle, Ellen Mowry, Rana Zabad, Bibi Bielekova, Nancy Monson, Cornelia Laule, Margaret Burnett, Teri Schreiner, Judith Grinspan, Ruth Dobson, Katerina Akassoglou, Jennifer Graves, Orla Gray, Penelope Smyth, Eva Kubala Havrdova, Jana Lizrova Preiningerova, Brenda Banwell, Naila Makhani, Claudia Lucchinetti, Georgina Arrambide, Elisabeth Maillart, Wendy Macklin, and Wendy Gilmore on behalf of the International Women in MS.

The information in the letter is the opinion of the consignees and may not necessarily reflect a policy of their institutions.

## Potential Conflicts of Interest

Nothing to report.

## References

[1] Moneim J , Coles A , Giovannoni G , et al. Ann Neurol 2018;84:329–330. 30063257 10.1002/ana.25306

[2] Bendels MHK , Muller R , Brueggman D , Groneberg DA . Gender disparities in high‐quality research revealed by Nature index journals. PLoS One 2018;13:e0189136. 29293499 10.1371/journal.pone.0189136 PMC5749692

[3] Shah S . Women in neurology: a WIN‐win. Ann Neurol 2018;84:165–167. 30014504 10.1002/ana.25290

[4] McDermott M , Gelb DJ , Wilson K , et al. Sex differences in academic rank and publications at top‐ranked US neurology programs. JAMA Neurol 2018;75:956–961. 29610899 10.1001/jamaneurol.2018.0275 PMC6142929

[5] Jagsi R , Griffith KA , Jones R , et al. Sexual harassment and discrimination experiences of academic medical faculty. JAMA 2016;315:2120–2121. 27187307 10.1001/jama.2016.2188 PMC5526590

[6] Hopkins N . Reflecting on fifty years of progress for women in science. DNA Cell Biol 2015;34:159–161. 25689304 10.1089/dna.2015.2803

[7] Coles A . Authorship of phase 3 trials in multiple sclerosis. Ann Neurol 2018;83:653–655. 29518276 10.1002/ana.25203

[8] Klein RS , Voskuhl R , Segal BM , et al. Speaking out about gender imbalance in invited speakers improves diversity. Nat Immunol 2017;18:475–478. 28418385 10.1038/ni.3707 PMC5775963

[9] Silver JK , Bank AM , Slocum CS , et al. Women physicians underrepresented in American Academy of Neurology recognition awards. Neurology 2018;91:e603–e614. 30030329 10.1212/WNL.0000000000006004 PMC6105044

[10] Moving forward. In: Seeking solutions: maximizing American talent by advancing women of color in academia: summary of a conference. 2013 Available at: https://www.nap.edu/read/18556/chapter/10. Accessed October 1, 2018.

[11] Data on women researchers in science . In: Blueprint for the future: framing the issues of women in science in a global context: summary of a workshop. 2012 Available at: https://www.nap.edu/read/13306/chapter/10. Accessed October 1, 2018.

[12] What to do when you realize the meeting you are speaking at is a YAMMM (yet another mostly male meeting)? Available at: https://phylogenomics.blogspot.com/2014/05/what‐to‐do‐when‐you‐realize‐meeting‐you.html. Accessed October 1, 2018.

